# Supplementary figures and images for: TYMV and TRV infect Arabidopsis thaliana by expressing weak suppressors of RNA silencing and inducing host RNASE THREE LIKE1
Source: PLoS Pathog. 2023 Jan 25;19(1):e1010482. doi: 10.1371/journal.ppat.1010482 (PMC9901757; doi:10.1371/journal.ppat.1010482)

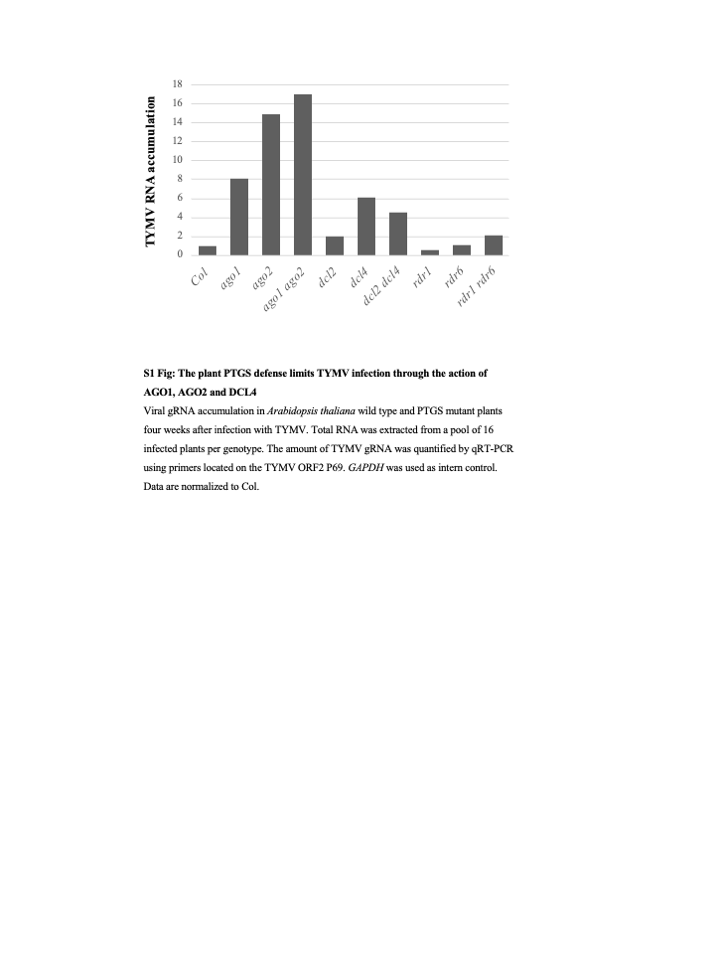

Supplement: S1 Fig — Viral gRNA accumulation in Arabidopsis thaliana wild type and PTGS mutant plants four weeks after infection with TYMV. Total RNA was extracted from a pool of 16 infected plants per genotype. The amount of TYMV gRNA was quantified by qRT-PCR using primers located on the TYMV ORF2 P69. GAPDH was used as intern control. Data are normalized to Col. (TIFF) [file ppat.1010482.s001.tiff]

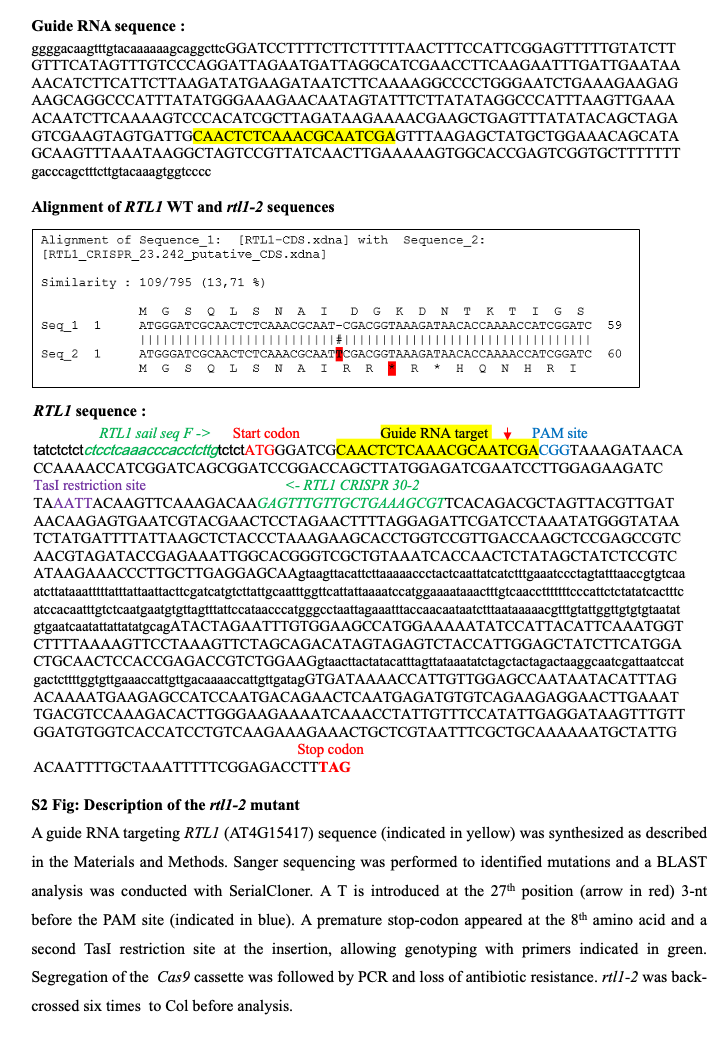

Supplement: S2 Fig — A guide RNA targeting RTL1 (AT4G15417) sequence (indicated in yellow) was synthesized as described in the Materials and Methods. Sanger sequencing was performed to identified mutations and a BLAST analysis was conducted with SerialCloner. A T is introduced at the 27th position (arrow in red) 3-nt before the PAM site (indicated in blue). A premature stop-codon appeared at the 8th amino acid and a second TasI restriction site at the insertion, allowing genotyping with primers indicated in green. Segregation of the Cas9 cassette was followed by PCR and loss of antibiotic resistance. rtl1-2 was back-crossed six times to Col before analysis. (TIFF) [file ppat.1010482.s002.tiff]
