# Supplementary material for: TYMV and TRV infect Arabidopsis thaliana by expressing weak suppressors of RNA silencing and inducing host RNASE THREE LIKE1
Source: PLoS Pathog. 2023 Jan 25;19(1):e1010482. doi: 10.1371/journal.ppat.1010482 (PMC9901757; doi:10.1371/journal.ppat.1010482)
Supplement: Attachment — Submitted filename: Responses to reviewers.docx [file ppat.1010482.s005.docx]

**Reviewer's Responses to Questions**

**Reviewer #1:**

Major issues:

1. The quality of viral infection data in Figure 1A should be improved. It looked like the plants were watered too much and not in good shape. And mock inoculated negative control should be shown. Biological repeats and statistics for analysis of viral RNA accumulation in different genotypes are lacking (Figure 1B).

In the original submission, the plants were properly watered, but the pictures we showed were taken too late. Indeed, plants were in good shape for about 20 days after infection, at least the ones that are not impaired in AGO1, AGO2 or DCL4. Those impaired in AGO1, AGO2 or DCL4 started to suffer after 20 days and eventually die from the infection. For this revision, we repeated the TYMV infection experiment twice and we now provide better pictures as well as quantitative data for the symptoms observed on the 13 genotypes analyzed in this study, which confirm our original conclusions. Unfortunately, we were not able to obtain a full set of RNAs of sufficiently good quality for either qRT-PCR or northern blot analysis in any of these two experiments. Nevertheless, the quantitative analysis of the symptoms shown in Figure 1 correlate with our previous qRT-PCR analysis so we are confident that this method, previously developed by the Carrington lab to quantify TCV infection (Zheng et al, 2019), is solid enough to support our conclusions. Because we have no replicate of the original qRT-PCR, we moved it as a supplemental data.

2. One evidence indicating that p69 inhibit RDR6 amplification is that p69 expression in Arabidopsis inhibit TAS3 siRNAs (Figure 4 B). As TAS3 siRNAs are dependent on miR390. It is necessary to compare the level of miR390 in absence and presence of p69. If p69 indeed inhibit RDR6 amplification step, miR390 mediated cleavage product of TAS3 should be still detected in both 6b4-306 and 6b4-306/p69 plants. Similarly, cleavage products containing GUS central sequences should also be detected in both genotypes.

Our RNA gel blot was rehybridized with a miR390 probe. P69 did not affect the accumulation of miR390, indicating that the reduction of TAS3 tasiRNA accumulation is not due to a reduction of miR390. We did not try to detect cleavage products. However, our data show that the transformation of the GUS central sequence (which is present in 6b4 target but not in the 306 inducer of primary siRNAs) into secondary siRNAs is inhibited by P69, and that the inhibition is proportional to the level of expression of P69 (GUS central siRNAs are reduced in pUBQ:P69 plants but absent in p35S:P69 plants)

3. Based on the subnuclear localization experiments in Arabidopsis roots and Nicotiana benthemiana leaves, p69 seems localized in differently in different organ, which may indicate it may function differently in these tissues. Analysis of GUS activity and small RNA accumulation in Arabidopsis roots or analyze p69 subcellular localization in Arabidopsis leaves is suggested to make a better ground for a conclusion about p69 function mechanism.

The localization experiments in nicotiana benthamiana leaves just aimed at confirming that our constructs were functional and that P69 behave in transient assay in leaves as previously described for transient assay in protoplasts. For co-localization experiments, we did not want to use transient assay so we generated stable transgenic P69 Arabidopsis, which we crossed with well characterized SGS3 and PAB2 lines. Because all the studies on siRNA-bodies and stress granules have been done on roots, we also used roots, which better material than leaves for subcellular localization by confocal microscopy. The aspect of the cytosol looks different in leaves and roots, but the localization of P69 is similarly diffuse in both tissues.

4. It is clear that RTL1 negatively regulates antiviral silencing against TYMV but not very convincing to conclude that RTL1 function in corporation with p69 to degrade amplified viral siRNAs based on just single rtl1 mutant phenotype. Comparison of viral siRNAs between rdr1/6 and rdr1/6/rtl1 may help test if RTL1 function through RDR mediated amplification step. An Y2H is also suggested to test whether RTL1 and p69 interact with each other.

It is true that a rdr1 rdr6 rtl1 triple mutant should show a better resistance to TYMV. However, we have not obtained this triple mutant yet. As for the 2YH experiment proposed by the reviewer, we do not understand what would be reason for P69 and RTL1 to interact with each other. Indeed, P69 does not inhibit RTL1 activity (Shamandi et al, 2015) so RTL1 could simply degrade RDR products (dsRNA) because P69 resides in siRNA-bodies where RDRs produce dsRNA. A Y2H screen was previously performed by Ni et al, 2017, and they did not retrieve RTL1 or any component of the siRNA-bodies. Therefore, it is possible that P69 partially inhibits the PTGS amplification step without interacting directly with its known components. For example, P69 could bind RNAs that are normally used as substrates by RDR6, thus reducing dsRNA production.

Importantly, in the revised manuscript, we show that the dual strategy used by TYMV to infect Arabidopsis likely is not a strategy unique to TYMV. Indeed, we found that a second virus, TRV, which, like TYMV, causes mild symptoms and, like TYMV, encodes a VSR that somehow impair the PTGS amplification step (Donaire et al, 2008), behave like TYMV with regards to RTL1, i.e. symptoms and amounts of viral genomic RNA are increased in *p35S:RTL1* plants and decreased in *rtl1* mutants. Therefore, this could be seen as a strategy commonly used by viruses expressing VSRs that only impair partially the plant anti-viral PTGS.

Minor issues:

1. Figure 4 B “GUS centrql” should be “GUS central”.

Corrected

2. Involvement of RTL1 in antiviral silencing is interesting, a picture of infected plants in comparison with wild type plants is suggested to show in the figure.

Images of infected rtl1 are now shown in Figure 6.

**Reviewer #2:**

Major issues

None.

Minor issues

- RQC needs to be explained.

We apologize for not explaining. This is now in a section of the introduction.

- Error bars are needed in Fig. 1B. This is critical, because the paragraph on lines 138 to 150 is based on comparisons between single and double mutants.

For the revision, we repeated the TYMV infection experiment twice so we now provide better pictures as well as quantitative data for the symptoms observed on the 13 genotypes analyzed in this study, which confirm our original conclusions. Unfortunately, we were not able to obtain a full set of RNAs of sufficiently good quality for either qRT-PCR or northern blot analysis in any of these two experiments. Nevertheless, the quantitative analysis of the symptoms shown in Figure 1 correlate with our previous qRT-PCR analysis so we are confident that this method, previously developed by the Carrington lab to quantify TCV infection (Zheng et al, 2019), is solid enough to support our conclusions. Because we have no replicate of the original qRT-PCR, we moved it as a supplemental data.Because the qRT-PCR in Fig6D is made from the same RNA as Fig1B, we had the same problem. We eventually decided to remove Fig6D because it is not essential for the paper.

- Line 144. AGO proteins do not produce primary siRNAs.

We apologize for the confusion. We have reworded as follows “Given that AGO1, AGO2 and DCL4 are sufficient execute PTGS using primary siRNAs”.

- Virus family and species need to follow standard nomenclature. Example, Potyvirus family is inaccurate.

We apologize for these mistakes. We have corrected this section according to the standard nomenclature.

- Both P19 and P38 are assigned to the Tombusviridae. Clarification is needed.

P19 and P38 are different VSR but come from two viruses of the same family (Tombusviridae). We have reworded the text so that it does not look like if all viruses of the same family encode the same VSR.

**Reviewer #3:**

Major issues

1. The writing of the manuscript is also very unsmooth. There are too many technical terms, which make me very difficult to read smoothly.

We apologize for the number of technical terms, but these are common in the RNA silencing field, and it is difficult to make it without them.

2. Fig 1A.The plants appear to have experienced severe environmental stress which may affect the disease symptom. In addition, an uninfected mock control is missing in all infection experiments.

The plants were properly grown. They were in good shape for about 20 days after infection, at least the ones that are not impaired in AGO1, AGO2 or DCL4. Those impaired in AGO1, AGO2 or DCL4 eventually die from the infection, and the pictures we originally showed were taken too late. For the revision, we repeated the TYMV infection experiment twice so we now provide better pictures as well as quantitative data for the symptoms observed on the 13 genotypes analyzed in this study, which confirm our original conclusions. Mock controls are shown.

3. To strength the authors’ conclusion that the inhibition of PTGS amplification depends on P69, I would suggest that authors to use a P69-deleted TYMV to perform virus infection assay in different RNA silencing mutants.

It is a very good suggestion. However, P69 is also the movement protein of the virus so plants do not propagate a P69-deleted TYMV (Bozarth et al, 1992). Nevertheless, we engineered a stop at the beginning of P69 by introducing the same mutation as described by Bozarth et al, 1992, and we cloned it in a p35S-expression vector (Pflieger et al, 2008). We inoculated both Col and rdr6 with p35S-TYMV and p35S-TYMV-stopP69. p35S-TYMV provoked an infection in both genotypes whereas p35S-TYMV-stopP69 did not, indicating that P69 actually is required for the movement of the virus.

4. Fig 1B and Fig 6D. The qPCR results lack error analysis and significance test of the difference. Moreover, to more accurately evaluate the accumulation of viral RNAs in plants, more experiments like RNA blotting assay should be performed.

Unfortunately, we were not able to obtain a full set of RNAs of sufficiently good quality for either qRT-PCR or northern blot analysis in any of the two new experiments we performed. Nevertheless, the quantitative analysis of the symptoms shown in Figure 1 correlate with our previous qRT-PCR analysis so we are confident that this method, previously developed by the Carrington lab to quantify TCV infection (Zheng et al, 2019), is solid enough to support our conclusions. Because we have no replicate of the original qRT-PCR, we moved it as a supplemental data.

Because the qRT-PCR in Fig6D was made from the same RNA as Fig1B, we had the same problem and could not repeat it. We eventually decided to remove this analysis because it is not essential for the paper.

5. Fig 5B. To better support the colocalization of P69 with secondary siRNAs in siRNA bodies, a colocalization of P69 with RDR6 should be provided.

SGS3 and RDR6 always colocalize in siRNA-bodies. Therefore, there is no reason to expect that P69 colocalize with SGS3 but not RDR6. Moreover, we would like to remind that P69 inhibit the production of secondary siRNAs, but we have never said that p69 was directly interacting with RDR6 or inhibiting RDR6. Indeed, a Y2H screen was previously performed by Ni et al, 2017, and they did not retrieve any component of the siRNA-bodies. Therefore, it is possible that P69 partially inhibits the PTGS amplification step without interacting directly with its known components. For example, P69 could bind RNAs that are normally used as substrates by RDR6, thus reducing dsRNA production.

6. Fig 6. TYMV infection symptom images of rtl mutants and viral RNA accumulation data should be provided.

Images are now shown. Viral RNA accumulation was already show and quantified in part B of Figure 6 (which is now part C of revised figure 6). We also show the data for p35S-RTL1 plants that were analyzed at the same time, which shows that inhibiting or overexpressing RTL1 have opposite effect.

Importantly, in the revised manuscript, we show that the dual strategy used by TYMV to infect Arabidopsis likely is not a strategy unique to TYMV. Indeed, we found that a second virus, TRV, which, like TYMV, causes mild symptoms and, like TYMV, encodes a VSR that somehow impair the PTGS amplification step (Donaire et al, 2008), behave like TYMV with regards to RTL1, i.e. symptoms and amounts of viral genomic RNA are increased in *p35S:RTL1* plants and decreased in *rtl1* mutants. Therefore, this could be seen as a strategy commonly used by viruses expressing VSRs that only impair partially the plant anti-viral PTGS.

Minor Issues

Line 180, What is QRC, Please explain.

We apologize for not explaining. This is now in a section of the introduction.

Fig 4B, The word “Centrql” should be “Central”.

Done
